# Supplementary material for: Joint Representation Learning for Retrieval and Annotation of Genomic Interval Sets
Source: Bioengineering (Basel). 2024 Mar 8;11(3):263. doi: 10.3390/bioengineering11030263 (PMC10967841; doi:10.3390/bioengineering11030263)
Supplement: Supplementary file 1 [file bioengineering-11-00263-s001.zip › bioengineering-2782260-supplementary.pdf]

Supplemental figures

Joint representation learning for retrieval and annotation of genomic interval sets

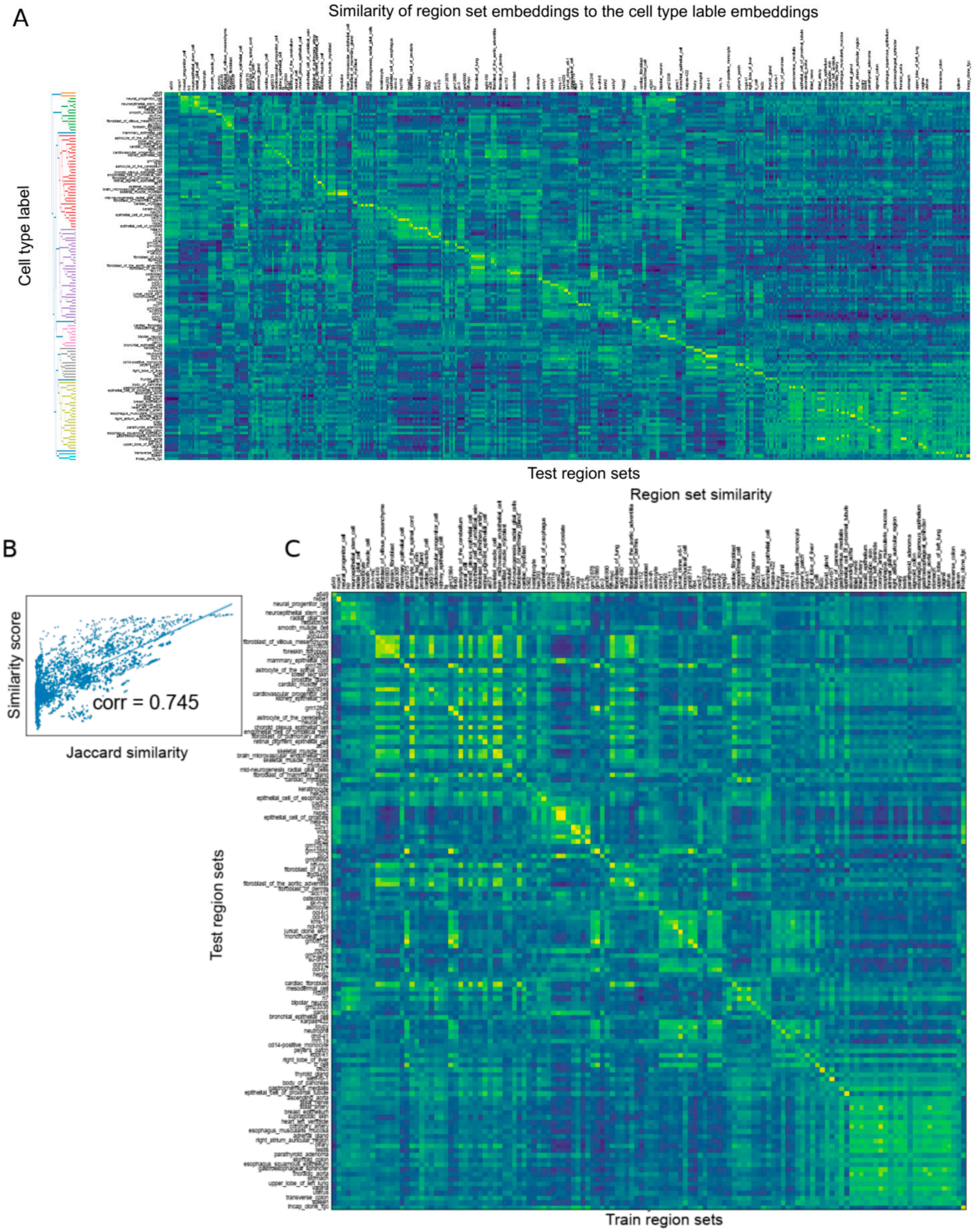

Supplementary Figure S1: Global performance trends. A) Heatmap showing the similarity between the cell type label embeddings and the embeddings of the region sets in the test dataset. The dendrogram depicts relationships among the label embeddings. B) The pair-wise similarity of a subset of BED file embeddings and their Jaccard similarity is correlated.C) The heatmap plot of the similarity between the test region set embeddings and the embeddings of the region sets in the training dataset. The model is trained on the cell type labels.

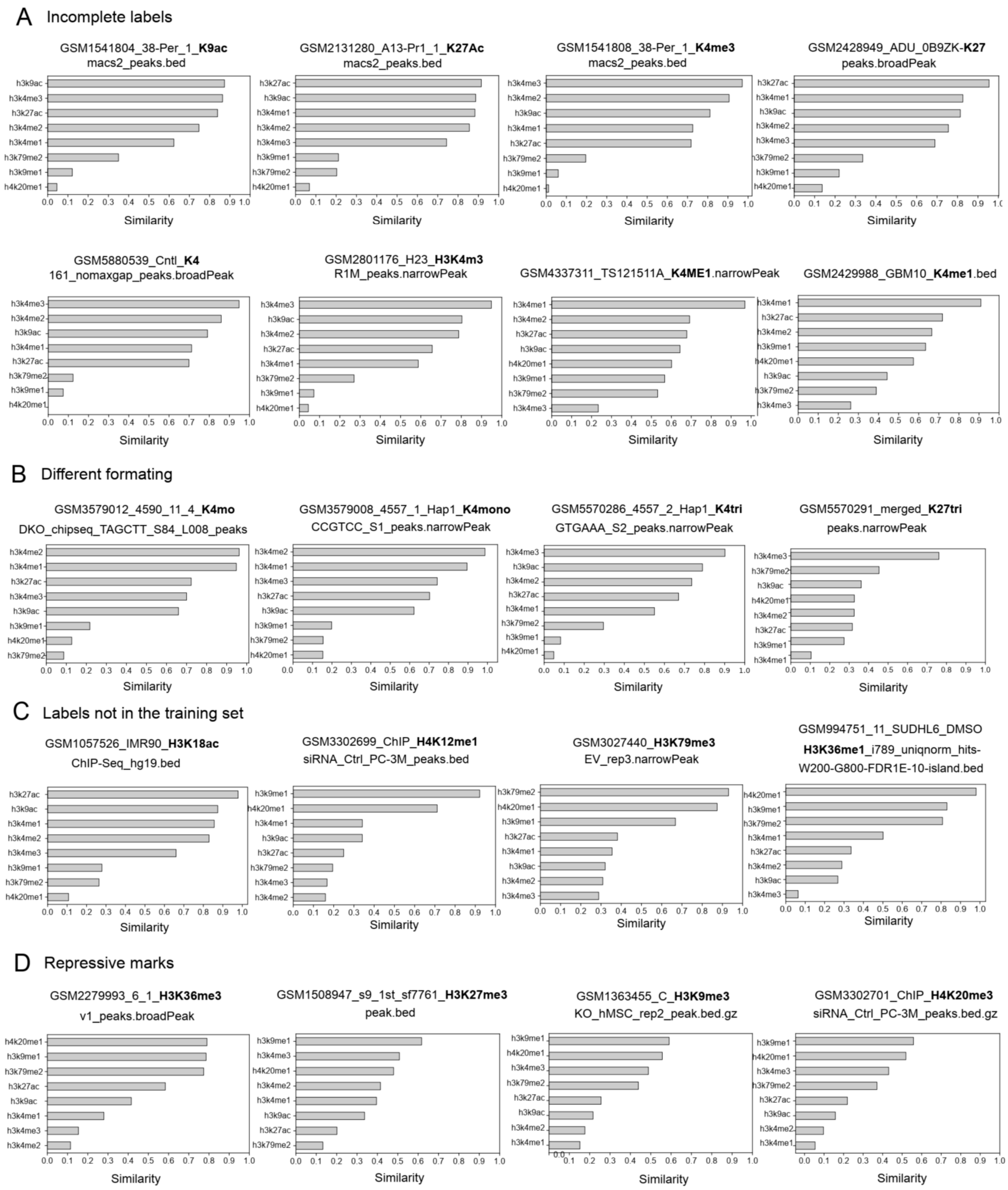

Supplementary Figure S2: Examples of corrected labels from public data sources. These examples show that the model can be used to A) complete the labels for files with incomplete labels; B) standardize metadata from different sources; C) identify the most related label when the label does not exist in the training model; and D) differentiate activating and repressive marks (for repressive marks, no high-confidence label is found).
